# Supplementary material for: Transcription-dependent targeting of Hda1C to hyperactive genes mediates H4-specific deacetylation in yeast
Source: Nat Commun. 2019 Sep 19;10:4270. doi: 10.1038/s41467-019-12077-w (PMC6753149; doi:10.1038/s41467-019-12077-w)
Supplement: Supplementary file 1 — Supplementary Information [file 41467_2019_12077_MOESM1_ESM.pdf]

Transcription-dependent targeting of Hda1C to hyperactive genes mediates H4-specific deacetylation in yeast

Ha et al.

Supplementary Figure 1

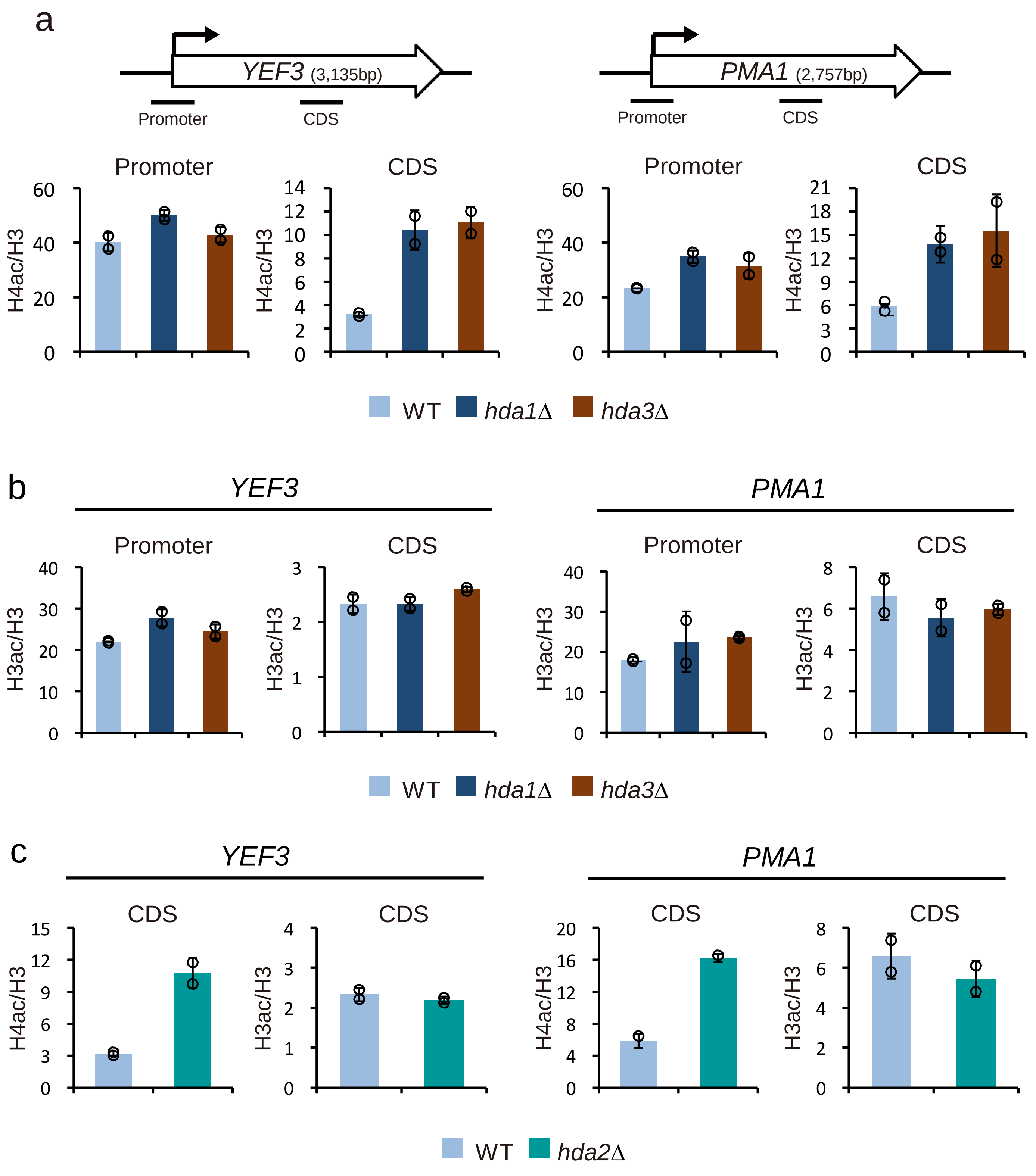

Supplementary Figure 1. Hda1C deacetylates H4 but not H3 at highly active genes.

(a) Loss of Hda1 and Hda3 results in increase of H4 acetylation at active coding regions. Crosslinked chromatin from the indicated strains grown in YPD was precipitated with anti-H3 or anti-acetyl H4 as indicated. ChIP assay was done as in **Fig. 1b**. (b) Hda1C is not required for H3 deacetylation at active genes. ChIP assay was done as in **a**. (c) Histone acetylation pattern within coding regions of *YEF3* and *PMA1* in *hda2*Δ. ChIP assay was done as in **a**. Source data are provided as a Source Data file.

Supplementary Figure 2

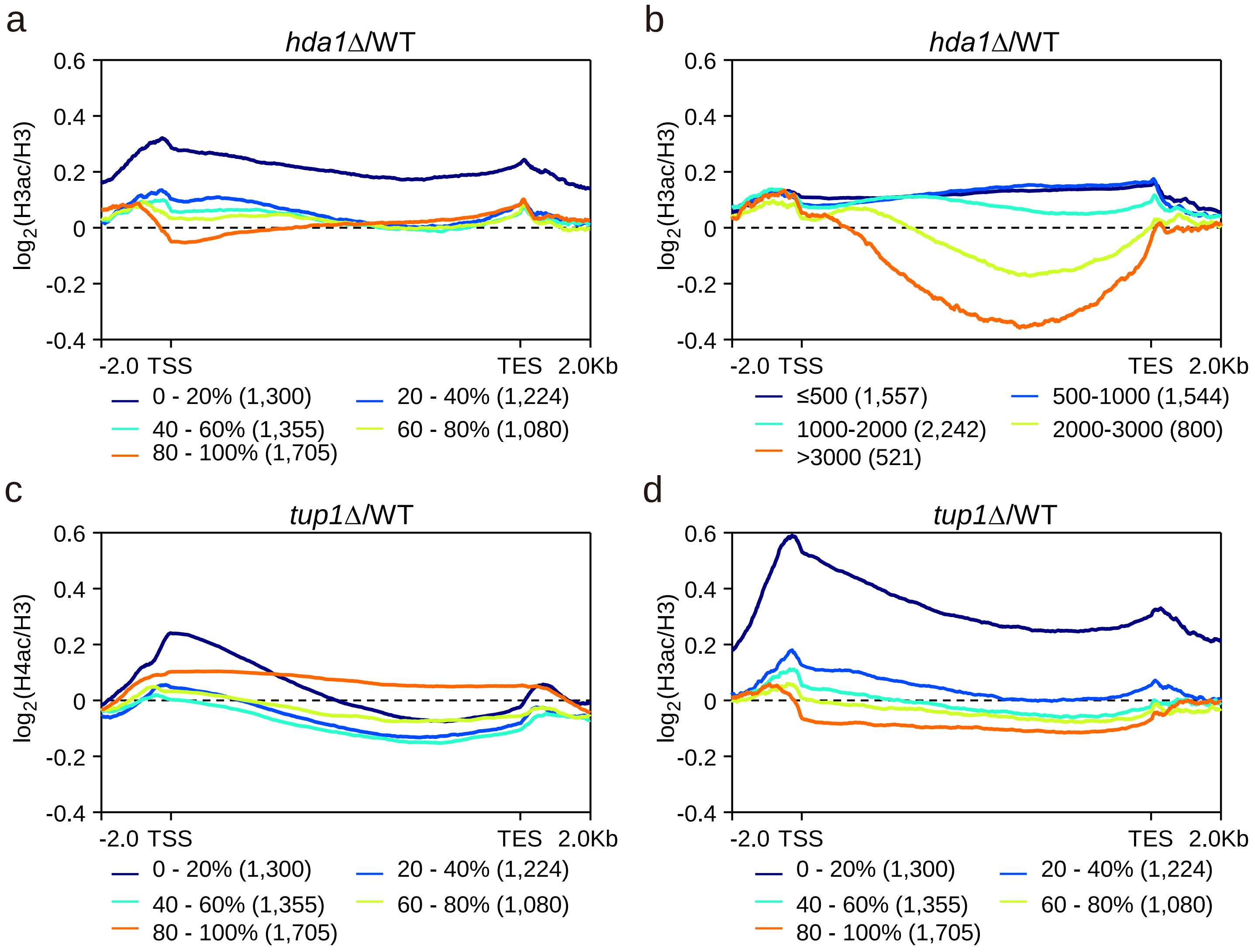

Supplementary Figure 2. Effect of *hda1*Δ or *tup1*Δ on global histone acetylation pattern.

(a-b) Inactive genes show an increase in H3 acetylation upon deletion of *HDA1*. Average plot of histone acetylation patterns based on RNA Pol II occupancy (Rpd3). Whereas the 0 - 20% group includes genes showing the lowest Rpb3 occupancy, the 80 - 100% group includes genes having the highest Rpb3 crosslinking (a). Average plot of H3 acetylation patterns based on gene length (b). (c-d) Tup1 is required for deacetylation of both histone H3 and H4. Average plot of H4 acetylation (c) or H3 acetylation (d) patterns based on RNA Pol II occupancy (Rpd3).

Supplementary Figure 3

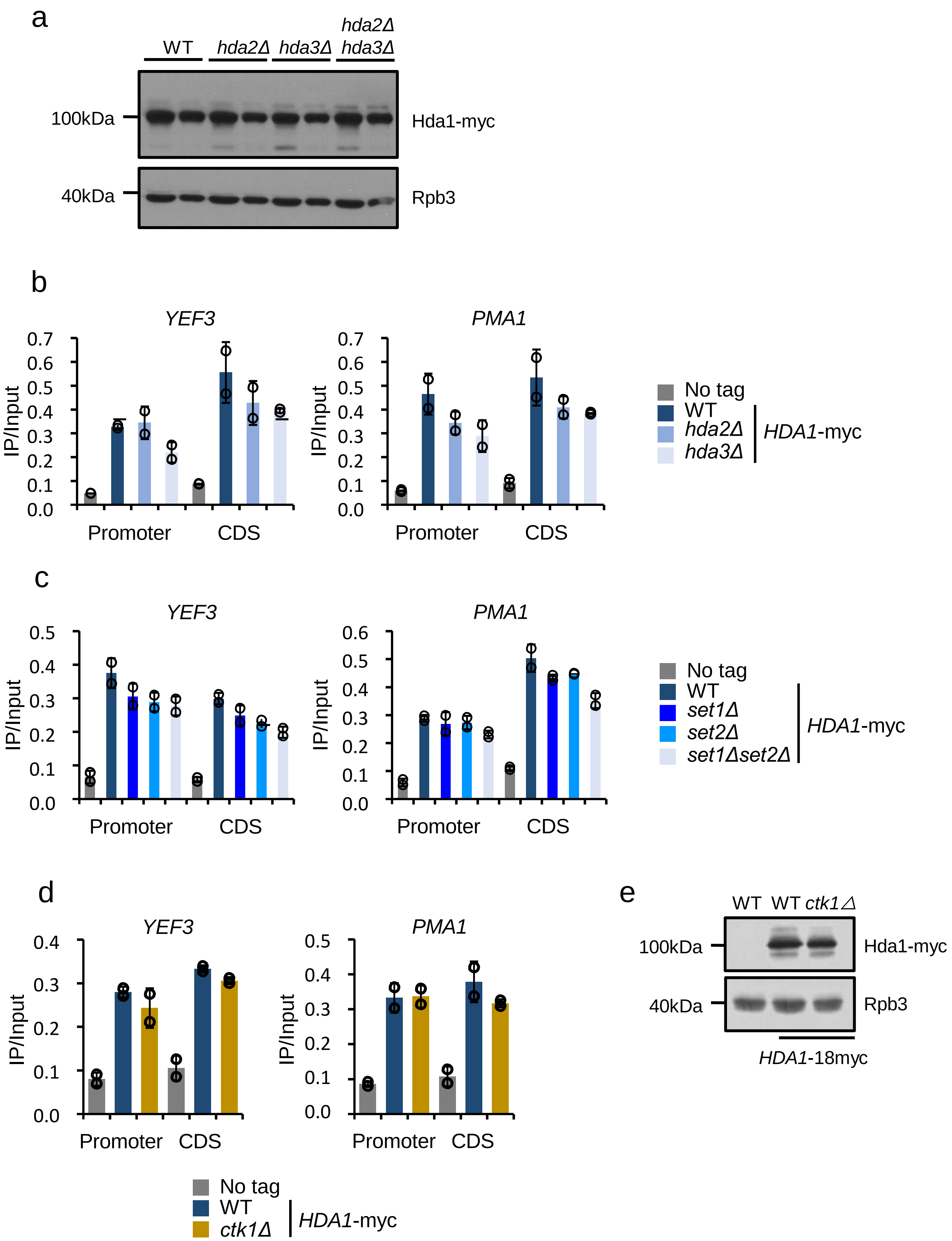

Supplementary Figure 3. Factors required for Hda1C recruitment.

(a) Loss of Hda2 or Hda3 does not affect Hda1 protein levels. Total proteins extracted from the indicated strains grown in YPD were separated by SDS-PAGE and probed with antibodies indicated on the right. Rpb3 was used as a loading control. (b) Hda2 and Hda3 partially affect Hda1 recruitment. ChIP assays using the indicated strains were performed as in **Fig. 3a**. (c) Loss of both Set1 and Set2 partially reduces Hda1 crosslinking. ChIP assay using the indicated strains was performed as in **Fig. 3a**. (d) Ctk1 is not required for Hda1C crosslinking. ChIP assay using the indicated strains was performed as in **Fig. 3a**. Source data are provided as a Source Data file. (e) Hda1 protein levels in wild-type and *ctk1*Δ cells. Western blot analysis was done as in **a**.

Supplementary Figure 4

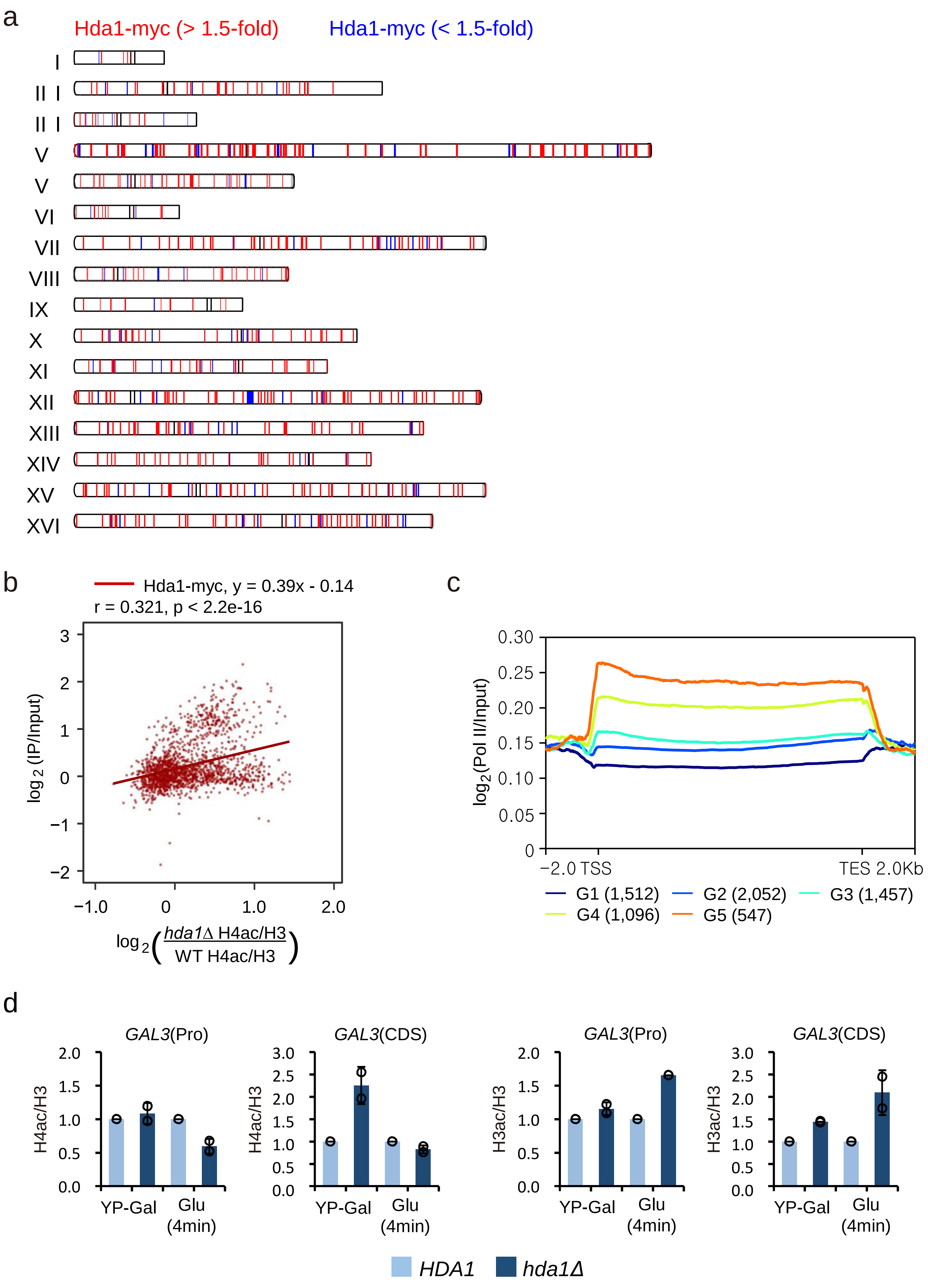

Supplementary Figure 4. Hda1C binds to highly transcribed genes to deacetylate histone H4.

(a) 571 Hda1C binding sites across yeast genomes. >1.5 fold (red) and <1.5 (blue). (b) The scatterplot shows a correlation between Hda1 binding and increase in H4 acetylation upon *HDA1* deletion at highly transcribed genes (top 25%) from **Fig. 4b**. Pearson's correlation coefficients are indicated. (c) Hda1C deacetylates histone H4 at highly active genes. Group 4 and 5 from **Fig. 2c** and **2d** showing a dramatic increase in H4 acetylation upon *HDA1* deletion exhibit relatively high levels of RNA Pol II. The y-axis indicates the normalized ChIP-seq reads and the x-axis indicates distance from the transcription start site (TSS) and transcription end site (TES). The number of genes is indicated in the parenthesis. (d) Crosslinked chromatin from the indicated strains, grown as described in **Fig. 4g**, was precipitated with an anti-H3, anti-acetyl H3, or anti-acetyl H4 antibody as indicated. PCR analysis of the precipitated DNA was performed as in **Fig. 1a**. Acetylation levels from wild type (*HDA1*) were set to 1. Source data are provided as a Source Data file.

Supplementary Figure 5

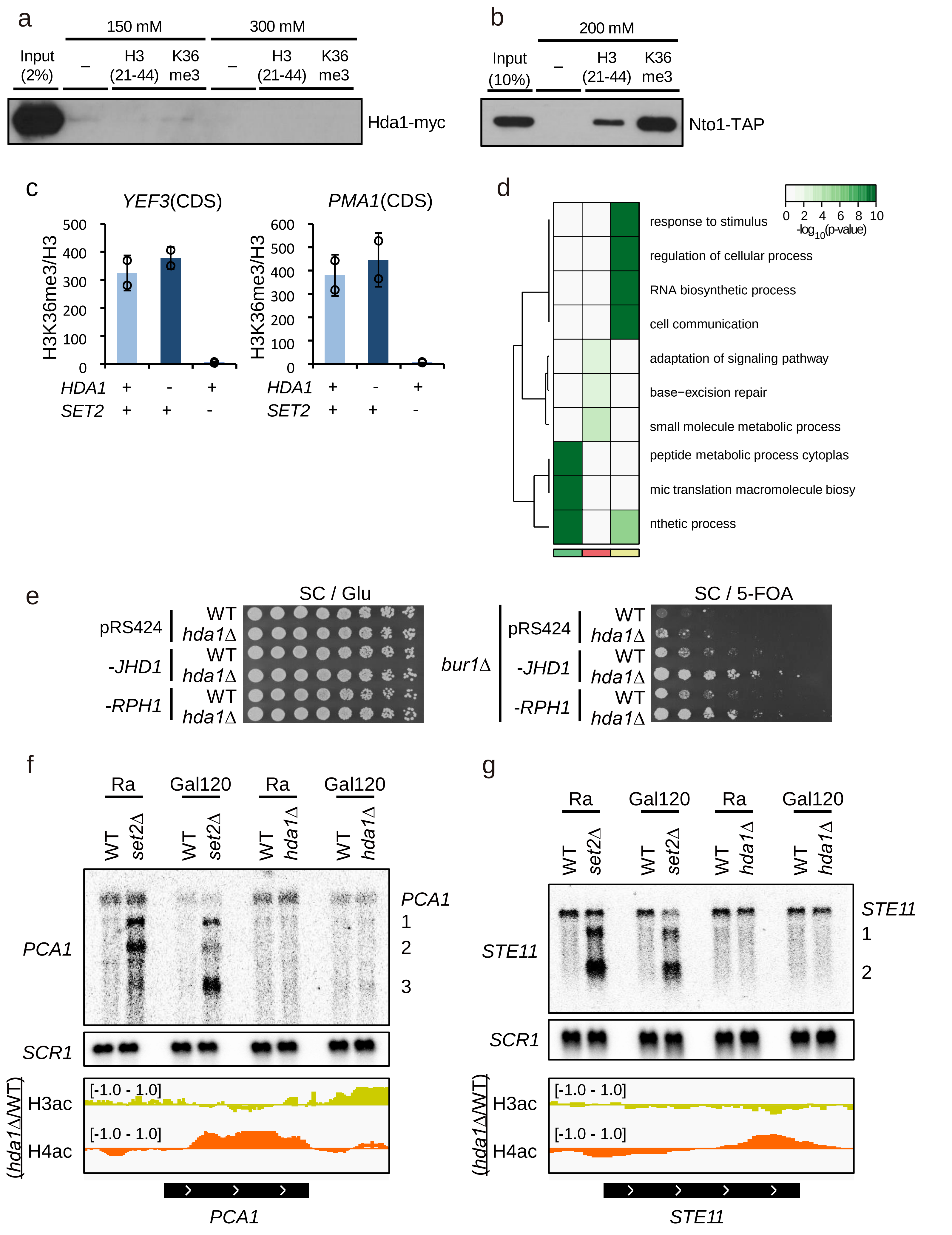

Supplementary Figure 5

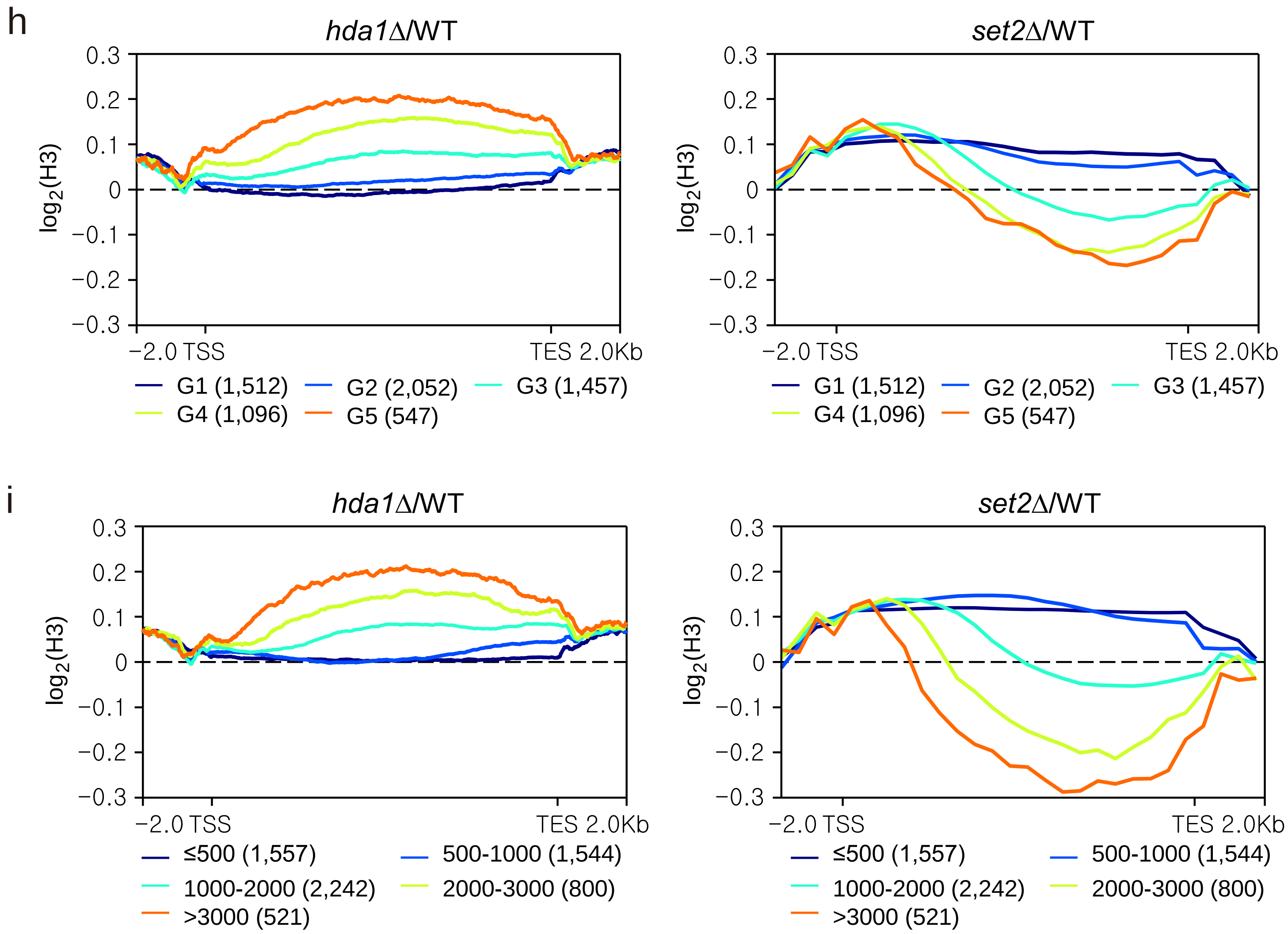

Supplementary Figure 5. Effects of H4-specific deacetylation by Hda1C.

(a) Hda1C does not bind to methylated K36 of histone H3. Histone peptide binding assay was performed with whole-cell extracts expressing Hda1-myc and 1 μg of the indicated histone peptides immobilized on magnetic beads. Precipitated Hda1 protein was analyzed by western blot analysis with anti-myc antibody. (b) Nto1, a subunit of NuA3 HAT strongly binds to methylated K36 of histone H3. Histone peptide binding assay was performed with whole-cell extracts expressing Nto1-TAP and 1 μg of the indicated histone peptides immobilized on magnetic beads. Precipitated Nto1 protein was analyzed by western blot analysis with anti-TAP antibody. (c) Loss of Hda1 does not affect H3K36me3 patterns. Crosslinked chromatin from the indicated strains grown in YPD was precipitated with anti-H3 or anti-H3K36me3 antibody. ChIP assay was done as in **Fig. 1b**. The *set2*Δ mutant was used as a control. Source data are provided as a Source Data file. (d) Hda1C and Set2 have distinct biological functions. Gene Ontology terms enriched in the three groups described in **Fig. 5d**. Significance was determined using hypergeometric tests. (e) Synergistic effects of overexpression of histone demethylases for H3K36 and loss of Hda1 on *bur1*Δ suppression. Jhd1 or Rph1 was overexpressed in the indicated strains. The resulting cells were spotted in 3-fold dilutions onto a SC/Glu plate (shown after 2 days) or a SC/Glu +5-FOA plate (shown after 8 days). (f-g) Hda1C does not affect cryptic transcription. The indicated strains were grown in SC-raffinose (Ra) and shifted to SC-galactose for 120min (Gal 120). *SCR1* is used as a loading control. (f) Northern blot analysis of *PCA1* cryptic transcripts (upper panel) and the log2 fold changes in H3 or H4 acetylation in *hda1*Δ cells versus wild- type cells (bottom panel). (g) Northern blot analysis (upper panel) and the log2 fold changes in H3 or H4 acetylation in *hda1*Δ cells versus wild-type cells (bottom panel). (h) Deacetylation of H4 by Hda1C may reduce histone occupancy. Average H3 occupancies in *hda1*Δ and *set2*Δ cells, categorized into the five groups shown in **Fig. 2c**. Whereas *hda1*Δ shows a slightly increased H3 levels within coding regions, the opposite pattern is seen in *set2*Δ. The number of genes is indicated in the parenthesis. (i) Longer genes show higher nucleosome occupancy than shorter genes upon deletion of *HDA1*. The plots show average H3 occupancies grouped by gene length. The number of genes is indicated in the parenthesis.

Supplementary Figure 6

a

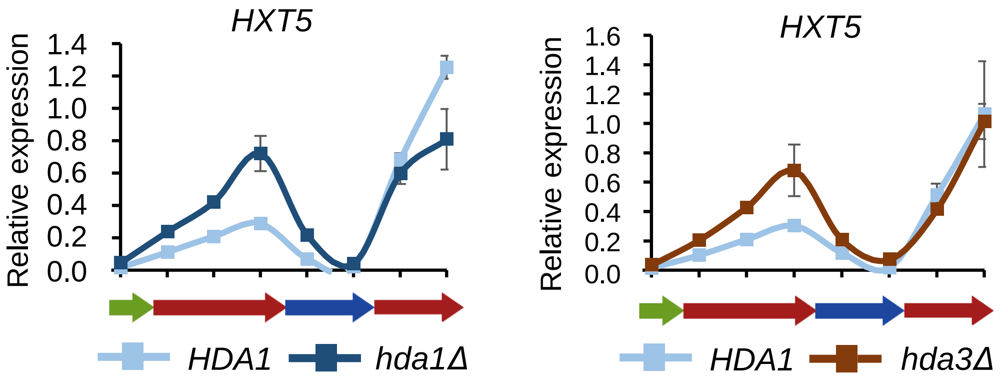

Supplementary Figure 6. Hda1C negatively regulates gene induction via H3 deacetylation.

(a) Expression patterns of *HXT5* in *hda1*Δ or *hda3*Δ during carbon source shifts. The time course experiments were done as **Fig. 6a**. The mRNA levels were determined by RT-PCR with two independent RNA samples. Error bars show the standard deviation (S.D.) calculated from two biological replicates, each with three technical replicates. Source data are provided as a Source Data file.

Supplementary Figure 7

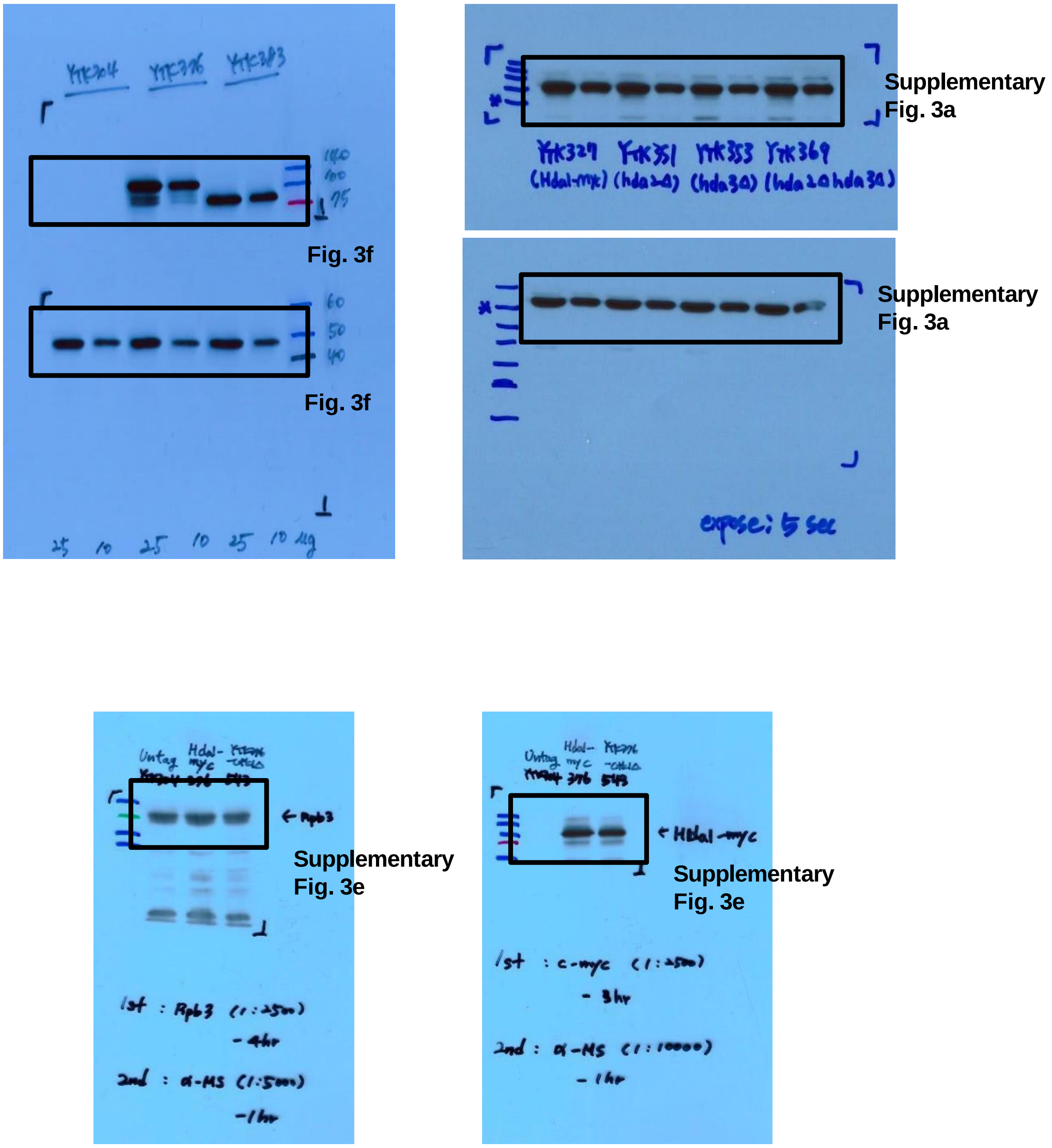

Supplementary Figure 7. Original western blots images for Fig. 3 and Supplementary Fig. 3. The uncropped scans of western blot exposed to x-ray films.

Supplementary Figure 8

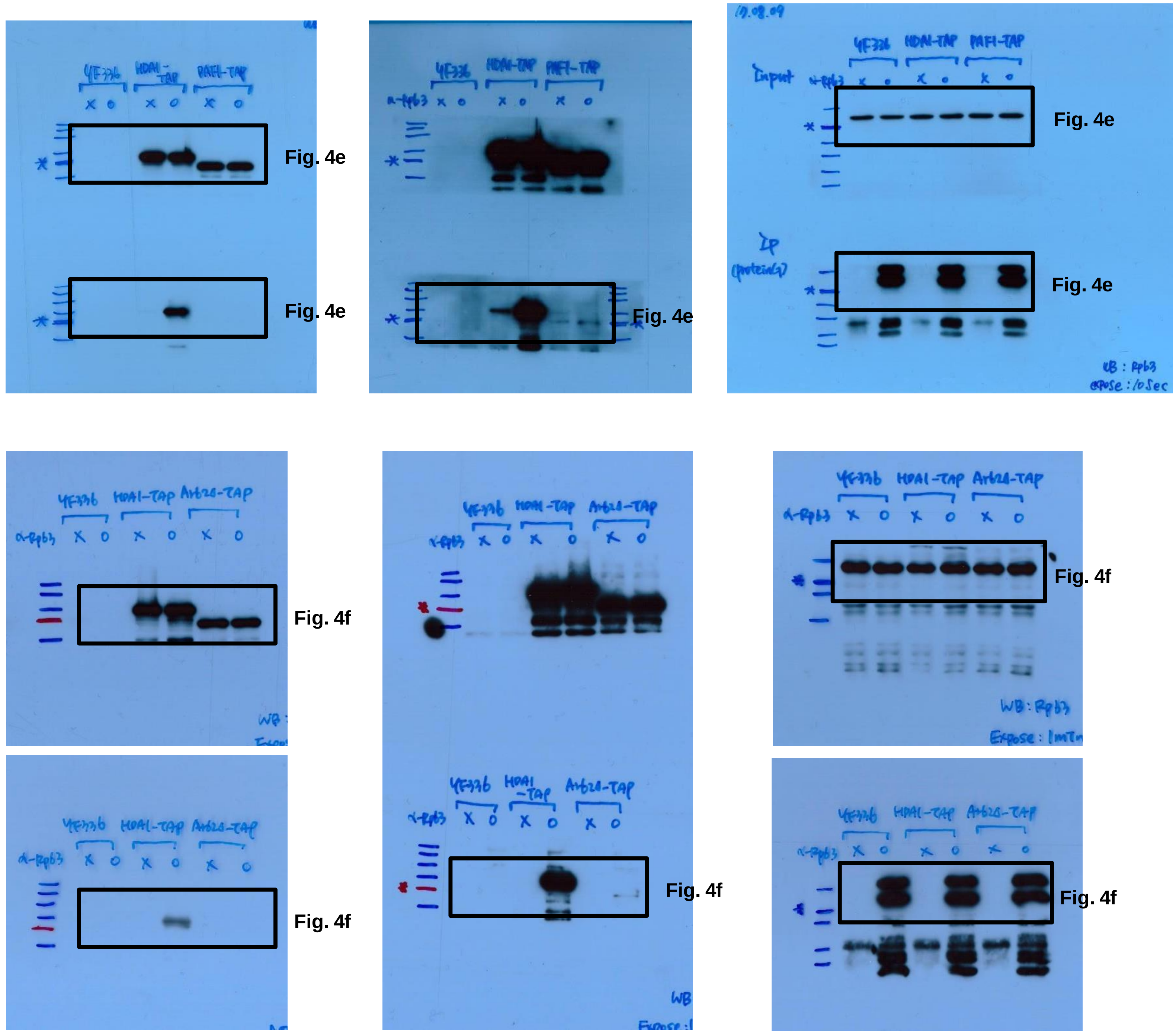

Supplementary Figure 8. Original western blots images for Fig. 4. The uncropped scans of western blot exposed to x-ray films.

Supplementary Figure 9

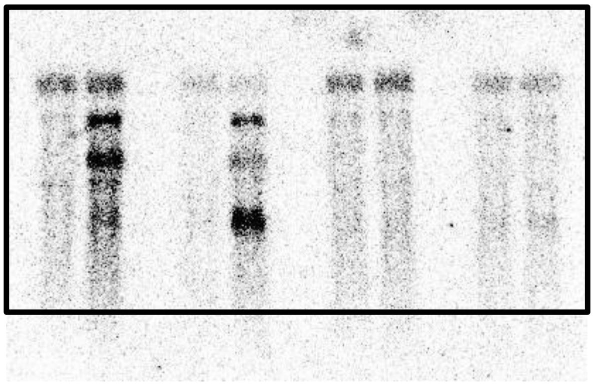

Supplementary Fig. 5h

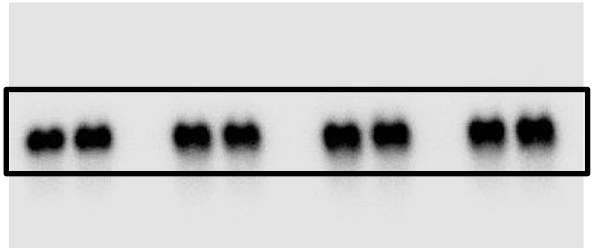

Supplementary Fig. 5h

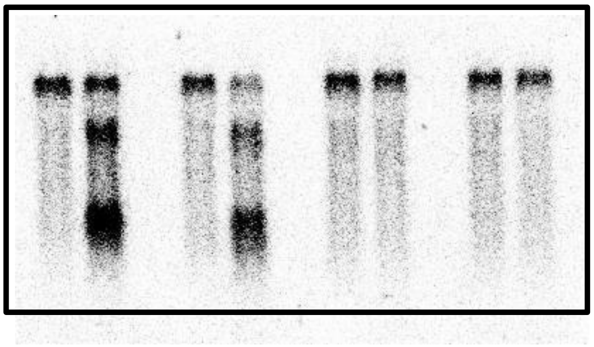

Supplementary Fig. 5i

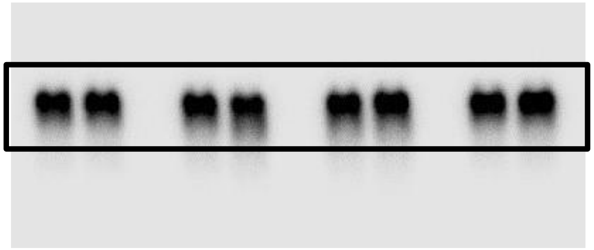

Supplementary Fig. 5i

Supplementary Figure 9. Original northern blots images for Supplementary Figure 5. The uncropped scans of northern blot exposed to x-ray films.

Supplementary Table 1. Strains used in this study.

Strains	Genotype	Source
BY4741(YF336)	MATa, *ura3Δ0, leu2Δ0, his3Δ1, met15Δ0*	Saccharomyces Genome Deletion Project
YSB787	MATa, *bur1Δ::HIS3, ura3-52, leu2Δ1, trp1Δ63, his3Δ200, lys2Δ202 (pRS316-BUR1)*	S. Buratowski
YSB1002	MATa, *bur1Δ::HIS3, ura3-52, leu2Δ1 or leu2Δ0, trp1Δ63, his3Δ200 or his3Δ1, lys2Δ202, met15Δ, set2Δ::KanMX4 (pRS316-BUR1)*	S. Buratowski
YSB2286	YF336-*set2Δ::NATMX*	S. Buratowski
YTK109	YF336-*hda1Δ::KanMX4*	This study
YTK367	YSB787-*hda2Δ::KanMX4*	This study
YTK111	YF336-*hda3Δ::KanMX4*	This study
YTK113	YSB787-*hda1Δ::KanMX4*	This study
YTK114	YSB787-*hda1Δ::KanMX4*	This study
YTK115	YSB787-*hda3Δ::KanMX4*	This study
YTK202	YSB787-*tup1Δ::KanMX4*	This study
YTK203	YSB787-*tup1Δ::KanMX4*	This study
YTK304	YF336-*trp1Δ::URA3*	This study
YTK327	YSB787- Hda1-18myc-*TRP1(K.lactis)*	This study
YTK329	YTK202- Hda1-18myc-*TRP1(K.lactis)*	This study
YTK351	YTK327-*hda2Δ::KanMX4*	This study
YTK353	YTK327-*hda3Δ::KanMX4*	This study
YTK369	YTK353-*hda2Δ::LEU2*	This study
YTK374	YF336-*HDA1*(ΔArb2 domain)	This study
YTK376	YTK304-Hda1-18myc-*TRP1(K.lactis)*	This study
YTK383	YF336-*HDA1*(ΔArb2 domain)-18myc-*TRP1(K.lactis)*	This study
YTK416	YTK376-*set1Δ::LEU2*	This study
YTK418	YTK376-*set2Δ::KanMX4*	This study
YTK430	YTK416-*set2Δ::KanMX4*	This study
YTK439	YSB2286(set2Δ::NATMX)-*hda1Δ::KanMX4*	This study
YTK543	YTK376-*ctk1Δ:: KanMX4*	This study
YF862	MATa, *ura3Δ0, leu2Δ0, his3Δ1, met15Δ0, PAF1-TAP tag::HIS3*	Nevan Krogan / Jack Greenblatt
YF1394	MATa, *ura3Δ0, leu2Δ0, his3Δ1, met15Δ0, HDA1-TAP tag::HIS3*	Yeast TAP-Fusion library(Open Biosystems)
YTK688	YTK374-*HDA1-TAP tag::HIS3*	This study
YFP1	Mat h- (972)	Daeyoup Lee

Supplementary Table 2. Oligonucleotides used in this study

Oligos	Purpose	Sequence
HDA1(Up156)_F	Knockout	TAGGTAAATAGAGCTGGGAG
HDA1(Dn150)_R	Knockout	GTAAAACACAGCTTAATGCATT
HDA1_Up_Leu2_F	Knockout	ATATTGAGAAAGGGAAAGTTGAGCACTGTAATACGCCGAACAGATTA AGC AACTGTGGGAATACTCAGGT
HDA1_Dn_Leu2_R	Knockout	CATAAGGCATGAAGGTTGCCGAAAAAAAATTATTAATGGCCAGTTTTT CC TACCCTATGAACATATTCCA
HDA3(up193)_F	Knockout	TACTGATTTAATCCACTCAGTT
HDA3(dn204)_R	Knockout	TGTATAGTAGTGATACGTGGT
TUP1_UpKanMX_F	Knockout	TTTTTTGATAAGCAGGGGAAGAAAGAAATCAGCTTTCCATCCAAACCA AT AGATTGTACTGAGAGTGCAC
TUP1_DnKanMX_R	Knockout	GAATAGTTTAGTTAGTTACATTTGTAAAGTGTTCCTTTTGTGTTCTGTT C CTGTGCGGTATTTCACACCG
TUP1(Up142)_F	Knockout	CCCCTCTATCTGTCCTTCTG
TUP1(Dn156)_R	Knockout	ATGGTGAGGAAAGTAACTGT
HDA1_PCORE_F(Arb2 del)	Knockout	CCTCCTGATGAACTACCTGATCCATTAAGTGATCCCAAGCCTGAGGT CAT GAGCTCGTTTTCGACACTGG
HDA1_PCORE_R(Arb2 del)	Knockout	GTTATCCCATAAATATATTAGAACTTCTTGGGATGTAATCATTCCTGAG T TCCTTACCATTAAGTTGATC
HDA1_Arb2_del_product	Knockout	ATGCGATTGCAAGAAGCGCTTTGAGTGTAGCAAAAGTACTAATAGGG GAG TACATCAAGTATTTTCCTTCTGTTGCCAAAATTGCTTTTATTGGAATAG G
SET1(Up200)_F	Knockout	ACTTGCGATTCTAGCTCTTT
SET1(Dn200)_R	Knockout	CTTCAACAGTGAGAGATCGC
SET2(Up130)_F	Knockout	GGTGGTTATTTATCAGAACG
SET2(Dn200)_R	Knockout	GTGTCACATTACCTATCATT
Hda1_18myc_F	myc tag	ACAGACTTTATACTGGATTCGTTTGAAGAATGGAGTGATGAAGAA TCCGGTTCTGCTGCTAGTGGT
Hda1_18myc_R	myc tag	GGCATGAAGGTTGCCGAAAAAAAATTATTAATGGCCAGTTTTTCC CCTCGAGGCCAGAAGACTAAG
TRP1del_URA3_F	Knockout	ATGTCTGTTATTAATTTCACAGGTAGTTCTGGTCCATTGG TTCAATTCAATTCATCATTT
TRP1del_URA3_R	Knockout	CTATTTCTTAGCATTTTTGACGAAATTTGCTATTTTGTTA CGCAGGGTAATAACTGATAT
HDA2_UpKanMX_F	Knockout	GGCTTCATTAGTGTGTGAAAAATAAAGAAAATAGATACAATACTATCG AC AGATTGTACTGAGAGTGCAC
HDA2_DnKanMX_R	Knockout	ATAAAATCTCTCTATATTATACAGGCTACTTCTTTTAGGAAACGTCACA T CTGTGCGGTATTTCACACCG
HDA2_Up_Leu_F	Knockout	GGCTTCATTAGTGTGTGAAAAATAAAGAAAATAGATACAATACTATCG AC AACTGTGGGAATACTCAGGT
HDA2_Dn_Leu_R	Knockout	ATAAAATCTCTCTATATTATACAGGCTACTTCTTTTAGGAAACGTCACA T TACCCTATGAACATATTCCA
HDA2(Up189)_F	Knockout	TAAAATAACATAATTGCGGCAC
HDA2(Dn192)_R	Knockout	GTTGATGAAATGTTTCTGAAGA
CTK1(up200)_F	Knockout	GGTTCAGACAGTAAAATACATTT
CTK1(dn200)_R	Knockout	GTGTTGGTACGATGGACAAA
HXT5_promoter_F	qRT-PCR	AGATTCGCTTTCACTTTGCA
HXT5_promoter_R	qRT-PCR	GTAGCAGACCCTTCCAAGGG
HXT5_CDS_F	qRT-PCR	CAAACTCTGTCCAATGGAGG
HXT5_CDS_R	qRT-PCR	TAGCTCTTGCTAAGGACCGC
SCR1_F	qRT-PCR	GAAGTGTCCCGGCTATAATAAA

SCR1_R	qRT-PCR	GACGCTGGATAAAACTCCCC
TKL2_promoter_F	qRT-PCR	AGTCGTATATTCAATTGGCT
TKL2_promoter_R	qRT-PCR	AATCTTAAAGTGGAAACCGC
TKL2_CDS_F	qRT-PCR	GAAGACTGTTGTGGAACCCG
TKL2_CDS_R	qRT-PCR	TTCGGTAAATGCTTTTCCCAAC
GAL1_promoter_F	qRT-PCR	ATGGAAAAGCTGCATAACCA
GAL1_promoter_R	qRT-PCR	AATCACTTCTTCTGAATGAG
GAL1_CDS_F	qRT-PCR	CTACTCCGTTTAAATTTCCG
GAL1_CDS_R	qRT-PCR	CAGCTAAAACATTTGCAGCT
GAL1_3'end_F	qRT-PCR	TACTGTTCACTTGGTTCCAG
GAL1_3'end_R	qRT-PCR	TCATATAGACAGCTGCCCAA
GAL3_promoter_F	qRT-PCR	TGCAACACATAGGCAGTAAA
GAL3_promoter_R	qRT-PCR	GTTGCCCTACCTTTTTACTT
GAL3_CDS_F	qRT-PCR	ATGAAATCAGTTTCGTCATC
GAL3_CDS_R	qRT-PCR	ATTGTCCTTGTGAGATGGTA
PMA1_promoter_F	qRT-PCR	GGTACCGCTTATGCTCCCCTCCAT
PMA1_promoter_R	qRT-PCR	ATTTTTTTTCTTTCTTTTGAATGTGTG
PMA1_CDS_F	qRT-PCR	CAGAGTTGTTGAAATCTTGC
PMA1_CDS_R	qRT-PCR	TGTCTGGAGGTCTTCAAAGC
YEF3_promoter_F	qRT-PCR	GCACGTGAAAAAGAAACGTTTTTAATG
YEF3_promoter_R	qRT-PCR	GATGTTACCATTCAAGAAAGAAGCGAC
YEF3_CDS_F	qRT-PCR	GGTTTGAAGTTGAGAAAGTACAAGGG
YEF3_CDS_R	qRT-PCR	TCAAAGTAGACTTACCAGCACC
TEL-VI_F	qRT-PCR	GCGTAACAAAGCCATAATGCCTCC
TEL-VI_R	qRT-PCR	CTCGTTAGGATCACGTTCGAATCC
HAP1_(ORF)_F	qRT-PCR	AAAAAGGCAAACCCTAGCA
HAP1_(ORF)_R	qRT-PCR	TCCTCCAAGTGTGTTTAAATC
STT4_(ORF)_F	qRT-PCR	GCATAAATTTGAACCACGGT
STT4_(ORF)_R	qRT-PCR	ATCTTAGTATCCTGGTTGCT
STE11_F	Northern probe	ATTCTGATGAGAATAATGAGC
STE11_R	Northern probe	AGAAATTCTTTCCTTCTGACG
PCA1_F	Northern probe	TGGAGTTCATATCAATGAGGGAA
PCA1_R	Northern probe	CATAACGCAGAAGTATAGCTACA
