# Supplementary material for: Transcription-dependent targeting of Hda1C to hyperactive genes mediates H4-specific deacetylation in yeast
Source: Nat Commun. 2019 Sep 19;10:4270. doi: 10.1038/s41467-019-12077-w (PMC6753149; doi:10.1038/s41467-019-12077-w)
Supplement: Supplementary file 3 — Reporting Summary [file 41467_2019_12077_MOESM3_ESM.pdf]

nature research

Corresponding author(s): TaeSoo Kim

Reporting Summary

Nature Research wishes to improve the reproducibility of the work that we publish. This form provides structure for consistency and transparency in reporting. For further information on Nature Research policies, see Authors & Referees and the Editorial Policy Checklist.

Statistical parameters

When statistical analyses are reported, confirm that the following items are present in the relevant location (e.g. figure legend, table legend, main text, or Methods section).

n/a	Confirmed	
☐	☒	The exact sample size (*n*) for each experimental group/condition, given as a discrete number and unit of measurement
☐	☒	An indication of whether measurements were taken from distinct samples or whether the same sample was measured repeatedly
☐	☒	The statistical test(s) used AND whether they are one- or two-sided<br>*Only common tests should be described solely by name; describe more complex techniques in the Methods section.*
☐	☒	A description of all covariates tested
☐	☒	A description of any assumptions or corrections, such as tests of normality and adjustment for multiple comparisons
☐	☒	A full description of the statistics including central tendency (e.g. means) or other basic estimates (e.g. regression coefficient) AND variation (e.g. standard deviation) or associated estimates of uncertainty (e.g. confidence intervals)
☐	☒	For null hypothesis testing, the test statistic (e.g. *F*, *t*, *r*) with confidence intervals, effect sizes, degrees of freedom and *P* value noted<br>*Give P values as exact values whenever suitable.*
☐	☒	For Bayesian analysis, information on the choice of priors and Markov chain Monte Carlo settings
☐	☒	For hierarchical and complex designs, identification of the appropriate level for tests and full reporting of outcomes
☐	☒	Estimates of effect sizes (e.g. Cohen's *d*, Pearson's *r*), indicating how they were calculated
☐	☒	Clearly defined error bars<br>*State explicitly what error bars represent (e.g. SD, SE, CI)*

Our web collection on statistics for biologists may be useful.

Software and code

Policy information about availability of computer code

Data collection

H3K36me3 ChIP-seq data (GSE61888) and Histone acetylation upon set2 deletion data (GSE28099) in the Gene Expression Omnibus (https://www.ncbi.nlm.nih.gov/geo). Rpb3 data (E-TABM-1033) in ArrayExpress (https://www.ebi.ac.uk/arrayexpress).

Data analysis

ballgown-2.4.2, BEDTools-2.26.0, Bowtie2-2.2.5, deepTools-2.3.5, DiffBind-2.2.3, FastQC-0.11.5, FASTX Toolkit-0.0.13, GOstats-2.40.0, Gviz-1.18.2, HISAT2-2.0.4, IGV-2.3.91, LIMMA-3.30.13, MACS-2.1.0, NOISeq-2.16.0, Picard-tools-2.6, QualiMap-2.2.0, R-3.4.1, Samtools-1.3.1, spp-1.10.1, StringTie-1.3.0, UCSCtoolkit were used to anlayse data.

ChIP-sequencing
Experiment and library preparation
Except for Fig. 1a and 3f-3g, all strains were grown in 200ml of YPD (Yeast extract Peptone Dextrose; 1% Yeast Extract, 2% Peptone, 2% Dextrose (Glucose)) at 30℃ until OD600 was 0.5-0.6. For Fig. 1a, the indicated strains were grown in YPD or YP-Galactose (1% Yeast Extract, 2% Peptone, 2% Galactose). For Fig. 3f-3g, cells were grown in YP-Galactose media to an optical density at 600nm of 0.6 and subsequently transferred to YPD. Cells were then subjected to 1% formaldehyde crosslinking. Chromatin immunoprecipitations were done using the following histone antibodies: anti-H3K36me3 (Abcam 9050), anti-acetyl H4 (Upstate 06-598), anti-acetyl H3(Millipore 06-599), anti-myc(BioLegend 626802) and anti-H3 (Abcam 1791). Precipitated DNAs were analyzed in real-time using THUNDERBIRD® SYPR® qPCR Mix (TOYOBO) and CFX96 cycler (Bio-Rad). ChIP-seq libraries were prepared using Accel-NGS 2S Plus DNA Library Kit (Swift Biosciences). The prepared libraries were sequenced on an Illumina HiSeq2500 using 100 bp paired end platform.

Alignment and quantification

Bowtie2-2.2.5 32 aligned sequences to the S. cerevisiae genome (R61-1-1). Picard-tools-2.6 (http://broadinstitute.github.io/picard) removed duplicates with parameters VALIDATION_STRINGENCY=silent AS=true. Information inferred from alignment was converted into a form of visualization by command genomeCoverageBed -bg -scale SCALE_FACTOR in BEDTools-2.26.0 34. IGV-2.3.91 35 is used for visualization of genomic tracks.

Comparison of enrichment between samples
The majority of processes in this subsection were performed with deepTools-2.3.5 36. Command bamCompare compared information of two files. Then, command computeMatrix calculated scores per genome regions and yielded a scoring matrix. There are two modes in this step; scale-regions was used to stretch all regions to the same length, and reference-point was used to focus on genomic positions before and after of the reference point. Finally, command plotProfile can plot the profile of metagenes from the scoring matrix. On the other hand, command plotHeatmap can plot the profile of individual genes. R function kmeans could divide individual genes in the scoring matrix into subsets with k-means clustering. GOstats-2.40.0 37 identified Gene Ontologies (GOs) enriched in certain genes. R-3.4.1 (http://www.r-project.org) was used to plot a heatmap with p-values of the result. Function plot of R-3.4.1 make a scatterplot and cor.test computed Pearson's correlation coefficients and P-values.

Identification of significantly enriched regions
MACS-2.1.0 39 identified peaks with command macs2 callpeak with minimum FDR (q-value) cutoff of 0.01. DiffBind-2.2.3 40 computed correlation of read counts in detected peaks between samples and identified the reproducibility between replicates. Gviz-1.18.2 41 visualized the location of the peaks on chromosomes.
Comparison of enrichment between samples
If the public data is raw data, limma-3.30.13 42 was used to process it. On the other hand, if the public data is processed data, it was directly used. IGV-2.3.91 35 is used for visualization of genomic tracks. Comparison of samples repeated the steps of ChIP-seq data analysis with deepTools-2.3.5 (Ramirez et al., 2016). Signals at the same probes between two samples were directly compared. Due to the low resolution of ChIP-chip data, scores in a scoring matrix were smoothened with windows of the size of 500 bp differently from ChIP-seq data with windows of the size of 10 bp.

RNA-sequencing
Total RNA was prepared by hot-phenol method. mRNA-seq libraries were prepared using NEXTflex Rapid Directional mRNA-Seq Kit (Boo Scientific). The libraries were sequenced on an Illumina HiSeq2500 100 bp paired end platform (Macrogen Inc., South Korea). HISAT2-2.0.4 (Kim et al., 2015) aligned sequences to the transcriptome (ENSEMBL 86) of S. cerevisiae (R61-1-1) with a parameter --rna-strandness RF. Information inferred from alignment was converted into a form of visualization by command genomeCoverageBed –bg -split -scale SCALE_FACTOR in BEDTools-2.26.0 (Quinlan and Hall, 2010). Reads on known transcript were counted by StringTie-1.3.0 with parameters -e -B 43. Fragments per kilobase of transcript per million mapped reads (FPKM) was used as a normalized expression level. Genes expressed with more than 1.7-fold in any comparison were regarded as significantly up-regulated genes. R-3.4.1 (http://www.r-project.org) was used to plot a heatmap of these genes. Reads on regions surrounding TSSs of these genes were counted. R-3.4.1 was used to draw a violin plot of the reads. R function permTS in perm package examined the difference between the counts by permutation test.

For manuscripts utilizing custom algorithms or software that are central to the research but not yet described in published literature, software must be made available to editors/reviewers upon request. We strongly encourage code deposition in a community repository (e.g. GitHub). See the Nature Research guidelines for submitting code & software for further information.

Data

Policy information about availability of data

All manuscripts must include a data availability statement. This statement should provide the following information, where applicable:
- Accession codes, unique identifiers, or web links for publicly available datasets
- A list of figures that have associated raw data
- A description of any restrictions on data availability

The RNA-seq and ChIP-seq datasets were deposited at Gene Expression Omnibus under accession number GSE121763.

Field-specific reporting

Please select the best fit for your research. If you are not sure, read the appropriate sections before making your selection.

☒ Life sciences ☐ Behavioural & social sciences ☐ Ecological, evolutionary & environmental sciences

For a reference copy of the document with all sections, see nature.com/authors/policies/ReportingSummary-flat.pdf

Life sciences study design

All studies must disclose on these points even when the disclosure is negative.

Sample size	All the experiments were done with at least two independent samples. Standard deviations were calculated from two biological replicates, each with three technical replicates.
Data exclusions	No data were excluded from the study.
Replication	The findings in this study were highly reproducible and all data were from two independent biological replicates, each with three technical replicates.

Randomization	Not relevant to this study as yeast cultures involve many of individual cells.
Blinding	Investigators were not blinded to group allocation.

Reporting for specific materials, systems and methods

Materials & experimental systems

n/a	Involved in the study
☒	☐ Unique biological materials
☐	☒ Antibodies
☐	☒ Eukaryotic cell lines
☒	☐ Palaeontology
☒	☐ Animals and other organisms
☒	☐ Human research participants

Methods

n/a	Involved in the study
☐	☒ ChIP-seq
☒	☐ Flow cytometry
☒	☐ MRI-based neuroimaging

Antibodies

Antibodies used	The following antibodies were used in this study.<br>anti-H3K36me3 (Abcam 9050)<br>anti-acetyl H4 (Upstate 06-598)<br>anti-acetyl H3(Millipore 06-599)<br>anti-myc (BioLegend 626802)<br>anti-H3 (Abcam 1791)
Validation	N/A

Eukaryotic cell lines

Policy information about cell lines

Cell line source(s)	All yeast strains used in this study are listed in Supplementary Table 1.
Authentication	All strains were verified by PCR of genomic DNA. For the strains expressing tagged proteins, the strains were verified first by PCR of genomic DNA and western blot analysis.
Mycoplasma contamination	Mycoplasma contamination is not an issue for yeast cultures.
Commonly misidentified lines<br>(See ICLAC register)	N/A

ChIP-seq

Data deposition

☒ Confirm that both raw and final processed data have been deposited in a public database such as GEO.

☒ Confirm that you have deposited or provided access to graph files (e.g. BED files) for the called peaks.

Data access links<br>*May remain private before publication.*	https://www.ncbi.nlm.nih.gov/geo/query/acc.cgi?acc=GSE121763<br><br>Token: ghczkcumzlwznil
Files in database submission	YSB787-1-H3ac_noDup.norm.bw<br>YSB787-2-H3ac_noDup.norm.bw<br>YSB787-1-H4ac_noDup.norm.bw<br>YSB787-2-H4ac_noDup.norm.bw<br>YSB787-1-H3_noDup.norm.bw<br>YSB787-2-H3_noDup.norm.bw<br>YTK113-1-H3ac_noDup.norm.bw<br>YTK113-2-H3ac_noDup.norm.bw<br>YTK113-1-H4ac_noDup.norm.bw<br>YTK113-2-H4ac_noDup.norm.bw<br>YTK113-1-H3_noDup.norm.bw

YTK113-2-H3_noDup.norm.bw
YTK202-1-H3ac_noDup.norm.bw
YTK202-2-H3ac_noDup.norm.bw
YTK202-1-H4ac_noDup.norm.bw
YTK202-2-H4ac_noDup.norm.bw
YTK202-1-H3_noDup.norm.bw
YTK202-2-H3_noDup.norm.bw
YTK304-1-IP_noDup.norm.bw
YTK304-2-IP_noDup.norm.bw
YTK304-1-Input_noDup.norm.bw
YTK304-2-Input_noDup.norm.bw
YTK376-1-IP_noDup.norm.bw
YTK376-2-IP_noDup.norm.bw
YTK376-1-Input_noDup.norm.bw
YTK376-2-Input_noDup.norm.bw
YTK383-1-IP_noDup.norm.bw
YTK383-2-IP_noDup.norm.bw
YTK383-1-Input_noDup.norm.bw
YTK383-2-Input_noDup.norm.bw
YSB787-1-H3ac_peaks.narrowPeak
YSB787-2-H3ac_peaks.narrowPeak
YSB787-1-H4ac_peaks.narrowPeak
YSB787-2-H4ac_peaks.narrowPeak
YTK113-1-H3ac_peaks.narrowPeak
YTK113-2-H3ac_peaks.narrowPeak
YTK113-1-H4ac_peaks.narrowPeak
YTK113-2-H4ac_peaks.narrowPeak
YTK202-1-H3ac_peaks.narrowPeak
YTK202-2-H3ac_peaks.narrowPeak
YTK202-1-H4ac_peaks.narrowPeak
YTK202-2-H4ac_peaks.narrowPeak
YTK304-1-IP_peaks.narrowPeak
YTK304-2-IP_peaks.narrowPeak
YTK376-1-IP_peaks.narrowPeak
YTK376-2-IP_peaks.narrowPeak
YTK383-1-IP_peaks.narrowPeak
YTK383-2-IP_peaks.narrowPeak
YSB787-1-H3ac_1.fastq.gz
YSB787-1-H3ac_2.fastq.gz
YSB787-2-H3ac_1.fastq.gz
YSB787-2-H3ac_2.fastq.gz
YSB787-1-H4ac_1.fastq.gz
YSB787-1-H4ac_2.fastq.gz
YSB787-2-H4ac_1.fastq.gz
YSB787-2-H4ac_2.fastq.gz
YSB787-1-H3_1.fastq.gz
YSB787-1-H3_2.fastq.gz
YSB787-2-H3_1.fastq.gz
YSB787-2-H3_2.fastq.gz
YTK113-1-H3ac_1.fastq.gz
YTK113-1-H3ac_2.fastq.gz
YTK113-2-H3ac_1.fastq.gz
YTK113-2-H3ac_2.fastq.gz
YTK113-1-H4ac_1.fastq.gz
YTK113-1-H4ac_2.fastq.gz
YTK113-2-H4ac_1.fastq.gz
YTK113-2-H4ac_2.fastq.gz
YTK113-1-H3_1.fastq.gz
YTK113-1-H3_2.fastq.gz
YTK113-2-H3_1.fastq.gz
YTK113-2-H3_2.fastq.gz
YTK202-1-H3ac_1.fastq.gz
YTK202-1-H3ac_2.fastq.gz
YTK202-2-H3ac_1.fastq.gz
YTK202-2-H3ac_2.fastq.gz
YTK202-1-H4ac_1.fastq.gz
YTK202-1-H4ac_2.fastq.gz
YTK202-2-H4ac_1.fastq.gz
YTK202-2-H4ac_2.fastq.gz
YTK202-1-H3_1.fastq.gz
YTK202-1-H3_2.fastq.gz
YTK202-2-H3_1.fastq.gz
YTK202-2-H3_2.fastq.gz
YTK304-1-IP_1.fastq.gz
YTK304-1-IP_2.fastq.gz

YTK304-2-IP_1.fastq.gz
YTK304-2-IP_2.fastq.gz
YTK304-1-Input_1.fastq.gz
YTK304-1-Input_2.fastq.gz
YTK304-2-Input_1.fastq.gz
YTK304-2-Input_2.fastq.gz
YTK376-1-IP_1.fastq.gz
YTK376-1-IP_2.fastq.gz
YTK376-2-IP_1.fastq.gz
YTK376-2-IP_2.fastq.gz
YTK376-1-Input_1.fastq.gz
YTK376-1-Input_2.fastq.gz
YTK376-2-Input_1.fastq.gz
YTK376-2-Input_2.fastq.gz
YTK383-1-IP_1.fastq.gz
YTK383-1-IP_2.fastq.gz
YTK383-2-IP_1.fastq.gz
YTK383-2-IP_2.fastq.gz
YTK383-1-Input_1.fastq.gz
YTK383-1-Input_2.fastq.gz
YTK383-2-Input_1.fastq.gz
YTK383-2-Input_2.fastq.gz

Genome browser session
(e.g. UCSC)

Please refer to '*._noDup.norm.bw' at 'https://www.ncbi.nlm.nih.gov/geo/query/acc.cgi?acc=GSE121763'

Methodology

Replicates

Duplicates per a condition

Sequencing depth

Paired-end with each pairs of 101 bp
Sample, Total reads, Uniquely aligned reads
YSB787-1-H3ac, 19,808,802, 15,162,570
YSB787-2-H3ac, 19,553,582, 15,338,579
YTK113-1-H3ac, 20,628,832, 15,847,808
YTK113-2-H3ac, 21,546,474, 16,561,565
YTK202-1-H3ac, 27,388,532, 20,319,558
YTK202-2-H3ac, 21,955,768, 13,324,274
YSB787-1-H4ac, 25,884,876, 22,813,064
YSB787-2-H4ac, 25,173,490, 22,157,991
YTK113-1-H4ac, 27,144,668, 23,975,125
YTK113-2-H4ac, 23,596,034, 20,917,033
YTK202-1-H4ac, 23,378,372, 20,876,467
YTK202-2-H4ac, 23,175,256, 20,455,748
YSB787-1-H3, 20,127,892, 18,411,394
YSB787-2-H3, 25,008,764, 23,097,206
YTK113-1-H3, 25,989,764, 23,707,424
YTK113-2-H3, 25,333,704, 23,230,352
YTK202-1-H3, 22,835,290, 21,090,234
YTK202-2-H3, 23,863,466, 22,012,651
YTK304-1-IP, 28,696,040, 21,590,151
YTK304-2-IP, 26,778,898, 22,181,918
YTK376-1-IP, 24,893,350, 21,434,043
YTK376-2-IP, 27,224,250, 23,584,863
YTK383-1-IP, 29,923,502, 21,998,687
YTK383-2-IP, 28,829,954, 20,503,539
YTK304-1-Input, 33,134,532, 29,252,642
YTK304-2-Input, 29,153,044, 25,448,525
YTK376-1-Input, 22,871,072, 19,790,876
YTK376-2-Input, 29,766,610, 23,981,522
YTK383-1-Input, 27,972,688, 26,137,234
YTK383-2-Input, 28,856,718, 25,319,097

Antibodies

Anti-Histone H3 (Abcam, ab1791), Anti-acetyl-Histone H3 (Millipore, Cat#06-599), Anti-acetyl-Histone H4 (Millipore, Cat#06-598), Purified anti-c-Myc (BioLegend, Cat#626802)

Peak calling parameters

macs2-2.1.0 with corresponding controls and a parameter -q 0.01

Data quality

At q-value 0.01, compared to corresponding H3
Sample, Number of peaks
YSB787-1-H3ac, 2,501
YSB787-1-H4ac, 2,897
YTK113-1-H3ac, 2,717
YTK113-1-H4ac, 2,753
YTK202-1-H3ac, 2,841

YTK202-1-H4ac, 3,183
YSB787-2-H3ac, 2,571
YSB787-2-H4ac, 2,928
YTK113-2-H3ac, 2,777
YTK113-2-H4ac, 2,792
YTK202-2-H3ac, 2,788
YTK202-2-H4ac, 3,197
At q-value 0.01, compared to corresponding input
Sample, Number of peaks
YTK304-1-IP, 40
YTK304-2-IP, 30
YTK376-1-IP, 862
YTK376-2-IP, 846
YTK383-1-IP, 573
YTK383-2-IP, 390

Software

FastQC-0.11.5 checked the quality of raw sequences. If necessary, FASTX Toolkit-0.0.13 was used filtered out reads with low qualities and mates were properly paired. Bowtie2-2.2.5 aligned sequences to the S. cerevisiae genome (R61-1-1). QualiMap-2.2.0 checked the quality of the alignment. Picard-tools-2.6 removed duplicates with parameters VALIDATION_STRINGENCY=silent AS=true. bigWig files were generated using command genomeCoverageBed -bg -scale SCALE_FACTOR in BEDTools-2.26.0; Scores represent RPM. narrowPeak files were generated using macs2-2.1.0 with corresponding controls and a parameter -q 0.01.
